# Patient acceptance and perceived utility of pre-consultation prevention summaries and reminders in general practice: pilot study

**DOI:** 10.1186/1471-2296-12-40

**Published:** 2011-05-26

**Authors:** Oliver R Frank, Nigel P Stocks, Paul Aylward

**Affiliations:** 1 Discipline of General Practice, School of Population Health and Clinical Practice, University of Adelaide, Adelaide, South Australia 5005, Australia

## Abstract

**Background:**

Patients attending general practices receive only about sixty per cent of the preventive services that are indicated for them. This pilot study explores patient acceptability and perceived utility of automatically generated prevention summary and reminder sheets provided to patients immediately before consultations with their general practitioners.

**Methods:**

Adult patients attending a general practitioner in a practice in Adelaide and a general practitioner in a practice in Melbourne, Australia for consultations in January and February 2009 received automatically-generated prevention summary and reminder sheets that highlighted indicated preventive activities that were due to be performed, and that encouraged the patient to discuss these with the general practitioner in the consultation. Patients completed a post-consultation questionnaire and were interviewed about their experience of receiving the sheets.

**Results:**

Sixty patients, median age 53 years (interquartile range 40-74) years, and 58% female, were recruited. Seventy eight per cent of patients found the sheets clear and easy to understand, 75% found them very or quite useful, 72% reported they had addressed with their general practitioner all of the preventive activities that were listed on the sheets as being due to be performed. A further 13% indicated that they had addressed most or some of the activities. 78% of patients said that they would like to keep receiving the sheets. Themes emerging from interviews with patients included: patient knowledge was enhanced; patient conceptions of health and the GP consultation were broadened; the consultation was enhanced; patient pro-activity was encouraged; patients were encouraged to plan their health care; the intervention was suitable for a variety of patients.

**Conclusions:**

Most patients reported that they found the prevention summary and reminder sheets acceptable and useful. The actual increase in performance of preventive activities that may result from this new intervention needs to be tested in randomised controlled trials.

## Background

Preventable diseases cause significant disability, premature deaths and generate large treatment costs [1]. Eighty five per cent of the Australian population attend a general practitioner (GP) at least once annually, making an average of six visits per year [2,3], providing many opportunities for GPs to offer and perform preventive activities. A seminal paper [4] highlighted the potential of opportunistically offering and performing preventive activities during consultations. General practitioners regard the provision of preventive care as a priority and as one of their major tasks [5-7], and patients welcome the opportunistic offering and performance of preventive services by their general practitioners (GPs) [8-10].

Ninety per cent of Australian GPs reported using computer systems in 2005 for medical record keeping [11,12], and it is likely that this proportion has risen since then. The use of clinical computer systems by GPs in various countries is high [13,14] or rising significantly [15]. This offers the possibility of improving care for much of the population through computer-based tools.

Reminding doctors opportunistically about care which is indicated for the patient has been found to have the largest and the most consistently positive effect of all strategies to change doctors' behaviour [16-25], but the increased efficacy of up to 24% has not been enough to achieve a level of 80% of patients having preventive activities performed within recommended intervals, which we believe is a realistic goal.

Despite the many strategies used to try to increase the performance of preventive services, patients in the USA and Australia receive only about 60% overall of indicated preventive services [26-35].

Of all the parties concerned with a person's health, it is the individual patient who has the greatest interest and who therefore may be expected to be the most motivated to maintain his or her health. Practical experience has taught that patients are often unsure about which preventive activities are indicated for them and how often each activity should be performed. Even if the patient knows which preventive activities are indicated and due to be performed, the classic Health Belief Model [36] proposes that although a person may perceive themselves as being at risk of illness, action to try to reduce that risk "will not occur unless a cue to action is present" [37]. The Protection Motivation Theory [37] highlights the importance of self-efficacy as a key factor. Three elements needed to activate patients to increase performance of preventive activities are: 1) educating patients about the risks to health and how to maintain health; 2) informing patients about their preventive health status; 3) providing a means for the patient to act readily on the information and advice [38,39].

A recent systematic review of 1,535 trials of reminders issued to clinicians about preventive services [19] included 26 studies in which patients as well as clinicians were reminded about preventive services, via "mail, phone, waiting room posters, home visits", or by "educating patients on the importance of preventive care to encourage return visits". The performance of preventive activities in those 26 studies was no greater than in the trials in which patients were not reminded, which suggests that the patient reminders used in those studies were ineffective.

The Health Belief Model and the Protection Motivation Theory suggest that giving patients personalised information and appropriate cues at a time when they can easily act on that information and advice may promote action. We decided to test the acceptability and the perceived utility to patients of educating and reminding them about indicated preventive services whilst they were waited to see their general practitioner (GP). The findings from this pilot study will inform a larger study that will examine effects of this new intervention on the performance of preventive services, and on other processes of care.

The aim of the study was to conduct a pilot of the new strategy of pre-consultation prevention summaries and reminders, to assess its acceptability and perceived utility to patients, and thereby its potential to increase the performance of preventive activities in general practice.

## Methods

### Setting

A general practice in Adelaide and a general practice Melbourne, Australia, with one GP in each practice participating in the study (OF in Adelaide and the author of the software that produced the prevention summary and reminder sheets, in Melbourne).

### Recruitment

#### Inclusion and exclusion criteria

Inclusion criteria: Patients aged 18 years and over who were attending for a consultation.

Exclusion criteria: Patients who appeared to the receptionist to be physically or mentally distressed, or who appeared unable to read the information sheet or sign the consent form.

The intervention was conducted in the Adelaide practice in January 2009, and in the Melbourne practice in February 2009. To avoid overloading practice staff, the sheets were offered only on some consulting days. Prevention summary and reminder sheets were generated at the beginning of chosen days for patients who had appointments on that day and who met the inclusion criterion. As they arrived for their consultations, patients who had no exclusion criteria were asked by the receptionist whether they would be willing to be involved in this study. If the patient agreed, the receptionist gave the patient a set of documents consisting of information about the study, a consent form, and the prevention summary and reminder sheet that had been generated for the patient. This continued until 60 patients had been recruited.

### Intervention

We developed a format for a patient prevention summary and reminder sheet that listed the preventive activities that were indicated for the patient, according to authoritative guidelines [40]. The sheet showed when each preventive activity had last been performed, the result or finding on that occasion, and when it had become or would become due to be performed again. For activities that were currently due to be performed, the sheet also briefly explained the benefit of undertaking the activity.

**Figure 1:**
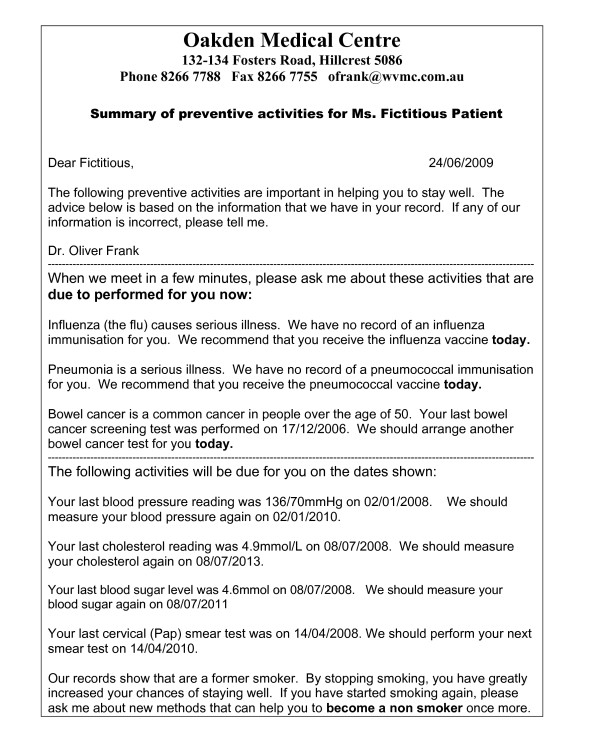
**Prevention summary and reminder sheet for a fictitious patient**.

Producing the sheets manually through employing a practice nurse for the task proved to be too time consuming and labour intensive for daily clinical practice. For efficiency, we decided to generate the sheets automatically using current data from each patient's electronic clinical record. As none of the clinical software packages available for Australian GPs were capable of generating and printing the sheets, we worked with the author of a freely-available add on clinical software package [41] to adapt that software for this purpose. An example prevention summary and reminder sheet generated through this collaboration appears in Figure 1.

Patients were asked to read the prevention summary and reminder sheet while waiting to see their GP and to take it in to the consultation with them. In the consultation, the patient could raise the issues identified on the sheets for discussion or action. If the patient did not mention the sheet or the issues listed on it, the GP was to provide usual care.

### Data collection

An independent field researcher interviewed participants by telephone two to three weeks after their consultation. The interview included Likert Scale questions to collate responses across specific indicators for the whole sample, and 'open-ended' questions to elicit opinions and experiences in greater depth and to embrace serendipitous findings. Statements to which responses were sought were:

• The information on the sheet was clear and easy for me to understand.

• I would like to keep receiving updated information sheets whenever I visit the GP. (Possible responses to these two statements were: 'strongly agree', 'agree', 'neither agree nor disagree', 'disagree', 'strongly disagree' or 'don't know').

• To what extent have you and your doctor addressed all of the things you've been advised to do on the sheet? (Possible responses were: 'all', 'most', 'some' or 'none').

• How useful was the information sheet to you? (Possible responses were: 'very useful', 'quite useful', 'not very useful' or 'not useful at all').

Participants were asked about their experience of receiving the prevention summary sheets, the influence this had had on the GP consultation and subsequent health behaviour. With their permission, the researcher recorded the interviews.

### Data analysis

Quantitative data were described by simple frequency count.

Thematic analysis of the qualitative replies was conducted independently by two of the researchers. Analytical interpretation drew from two key stages in grounded theory, open coding and categorisation. Through open coding, patient answers were read and re-read in order to identify and label each discrete idea and concept raised. Where labels coincided, comparisons were made both within individual patient responses and between patients and their meanings revised into thematic categories. Categories produced by each researcher were explored and revised and the revised categories compared against the original data to ensure they reflected the meanings in the patient responses. The six final themes were constructed through this process.

### Ethics approval

Ethics approval was given by the Human Research Ethics Committee of the University of Adelaide.

## Results

### Quantitave data

Sixty of 70 patients from the two practices who were invited to participate agreed (recruitment rate 86%), with 35 patients recruited in the Adelaide practice and 25 patients recruited in the Melbourne practice. Receptionists in the participating practices reported that they excluded few patients on grounds of patient distress or inability to read the sheet. Participating patients' median age was 53 years (interquartile range: 40-74 years) and 58% were female. The field researcher was able to contact 53 of the 60 (88%) patients for interview.

**Figure 2:**
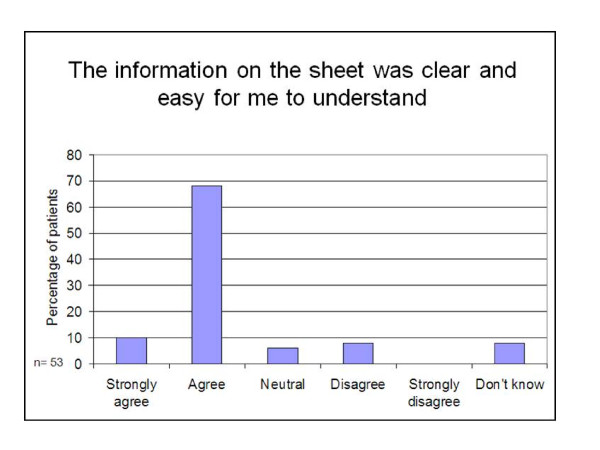
**Clarity of information and ease of understanding it**.

**Figure 3:**
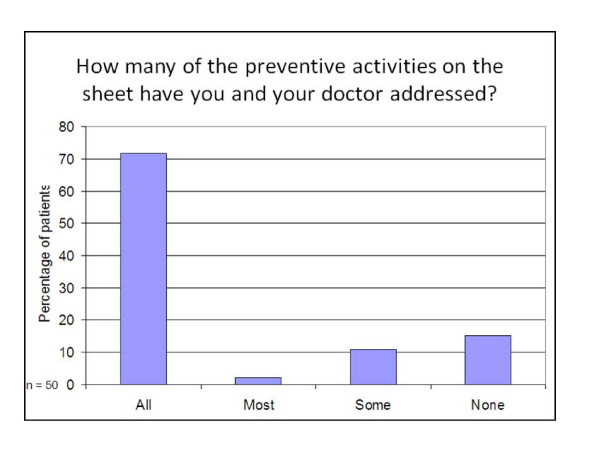
**Proportions of preventive activities addressed**.

**Figure 4:**
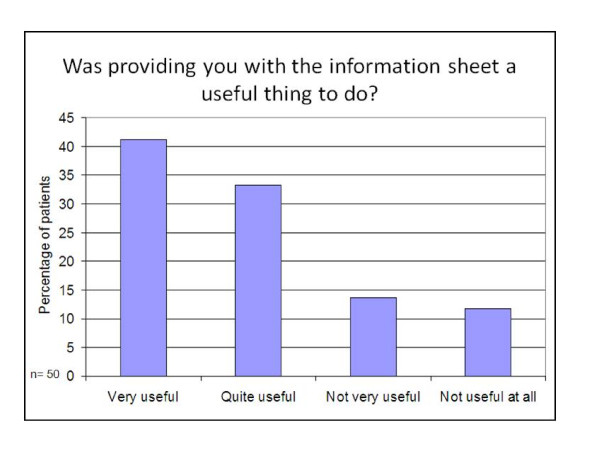
**Utility of the prevention summary and reminder sheet**.

**Figure 5:**
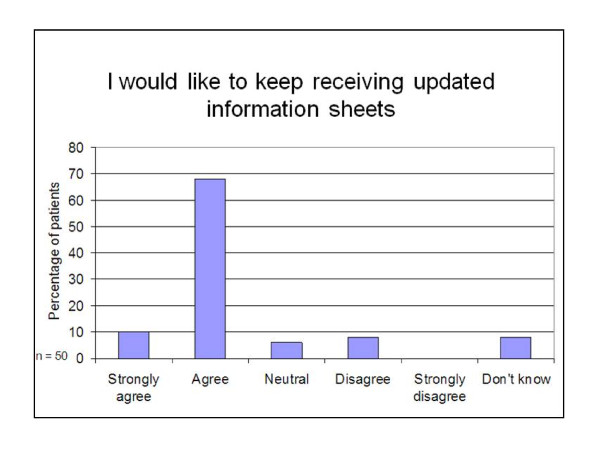
**Patients' wishes about receiving prevention summary and reminder sheets in the future**.

Responses from the quantitative 'closed' questions asked in the interviews are shown in Figures 2,3,4 and 5. Patients reported that they had found the information on the sheets clear and easy to understand (78% of participants), had addressed some or all of the preventive activities that were due (85% of participants), had found the sheets useful (75% of participants) and wanted to keep receiving them in the future (78% of participants).

### Qualitative data

The themes identified by the two researchers in the qualitative data were found to be highly congruent. Six related themes emerged from the interviews:

#### 1. Enhanced patient knowledge

Patients highlighted that the information sheets helped raise their understanding and knowledge:

"I am a smoker and the doctor gave me information about harmful effects of smoking - some things I didn't realize how harmful it is. I know what I have to do now." Participant H5, female, 33 years.

"Yes it gave me information that I didn't know - for example the injection I can have to stop me getting pneumonia - because I have bad chest infections in winter - and I didn't know I should still be having smear tests." M23, female, 59 years.

#### 2. Broadening patient conceptions of health and the GP consultation

The intervention has encouraged patients to consider their health and the consultation with their GP in broader preventive terms. This has encouraged a more comprehensive consultation addressing patient needs which otherwise may have been overlooked. Moreover, raising awareness of the range of preventative measures has stimulated patients to re-consider their use of GP services:

"A lot of people just go for a sick note or a cold. I used to be like that. I think it's changed that for me. It prompts you and reminds you to have checks. You might find something wrong but you can get it early." H17, female, 29 years.

"It brought up other things - usually I go to the doctor for a particular thing, but this time I caught up on everything." M2, female, 90 years

#### 3. Enhancing the consultation

Patients highlighted that the sheet stimulated discussion with their GPs which enhanced the consultation for both parties. This was viewed as promoting joint management of their health:

"Putting things down on paper makes it easier to talk because it's not so personal so you can talk about it in a general way. If it happens to you it's not so easy to talk because it's happening to you." H20, female, 20 years.

"I think this is good for GPs - too many times they do a few notes on a piece of paper, and continuity seeing the same doctor is sometimes a problem, and you feel ongoing health is not in mind. But you feel as though someone is looking after you. This sheet provides an extra level of service and these clinics probably will have better relationships with their patients too." M8, female, 90 years.

"It helps you get more out of the consultation It's a useful way to spend the time while you're waiting If I have to wait 15 minutes to see the doctor I would much rather read this than the "Women's Day" or the news. It reinforces the consultation." H32, male, 37 years.

#### 4. Encouraging patient pro-activity

The information sheets serve as a reminder to patients of when action is due. Moreover, raising awareness of health has encouraged patients to be more pro-active:

"It helped because it raised awareness about things and prompted me to ask about things I hadn't thought about before." H5, female, 33 years.

"I bet there's a whole lot of blokes out there just like me that don't care a hoot (about their health) unless something like this is put in front of them." H29, male, 53 years.

#### 5. Encouraging patients to plan their health care

The prevention summary sheets have encouraged patients to plan their future care. This has encouraged them to take greater control over the management of their health and creates the potential to optimise their GP consultation:

"It was a different approach. It was a useful prompt because I rarely visit the doctor and I sometimes don't think into the future about health." M15, female 32 years.

"At home I can refer to this sheet after the appointment at home and plan the next consultation and what I might talk about next time." H32, male, 37 years.

#### 6. Suitability for a variety of patients

Patients indicated that the prevention summary sheets were a suitable intervention for a broad range of patients. This has important implications for the broader implementation of the intervention:

"It would help people in age group 16-30 because older people are more aware of their health but younger people don't realise that they should have health checks too." H20, female, 20 years.

"Middle aged people in particular, because that's when their body and lifestyle changes most and they need to be more aware." M4, female, 26 years.

### Comments outside these themes

Several patients said that the high quality of service for their specific issues, or their own good knowledge of their health care needs, made the sheets not useful for them, but where these comments were made they were typically accompanied by positive feedback, and these patients said that the sheets were useful for others. One patient said that he was not ready to stop smoking. For some patients, while the sheet was welcomed, the recruitment procedures in place for the study reduced the time available to read the information sheet before seeing their GP.

Two additional limitations were cited: that the sheet being written in English was difficult for migrant people who could not read very well; in one case some of the information on the sheet was incorrect.

No patients said that the prevention summary and reminder sheets were unacceptable, could cause harm or adversely affect their relationship with their GP or practice.

## Discussion

This study found that most patients reported that they found the prevention summary and reminder sheets acceptable and useful, that they acted on the advice in them, and that they wished to keep receiving them in the future.

We have not found any studies in which individualised preventive care summaries and/or reminders were given or sent to patients who had already made appointments at general practices. Studies in which letters with preventive care advice were sent to patients of general practices who had not already made appointments found a poor response [38,39]. Waiting room posters or brochures present information and advice opportunistically, but the information and advice presented is not specific to the patient. Patient-specific information sent to patients who have not already planned to attend the practice requires patients to make an appointment solely for this purpose, requiring extra effort and time and often incurring extra cost [33,34]. Opportunistic reminders presented to doctors on paper or on screen compete with other tasks on the doctor's agenda, and are often not acted upon [14-17].

We believe that patients responded so positively to the intervention because it encouraged and enabled them to request and receive indicated preventive services and in many cases to receive those services immediately, with minimal additional time, effort or financial cost [10].

Our study had three main limitations that may have influenced the results. Firstly, funding and reporting deadlines made it necessary for us to recruit patients and deliver the intervention at the same visit, which caused us to provide several pages of information about the study, as well as the prevention summary and reminder sheet, at the recruitment visit. While this could have limited the ability of patients to read and respond to the information on the prevention summary and reminder sheet, only a minority of patients reported this. The problem would be considerably reduced should the procedure be adopted in routine practice. Secondly, we conducted the study in only two practices which had also been involved in the design of the information sheets. This could limit the generalisability of the study results. However, we were interested in the acceptability of the intervention, and because participants were broadly representative of the Australian population we feel that we obtained sufficient diversity of opinion. Lastly, some patients did not participate, which could lead to bias if non participants differed systematically from participants. Because only a few patients were excluded or declined to participate, we feel that the likelihood of this is low. Anecdotally no concerns were raised regarding the procedures for generating and distributing the sheets to patients, and few patients were excluded by the receptionists on grounds of illness or distress. The GPs involved found that the sheets enhanced the patient consultation. No concerns were expressed by patients or GPs about increased duration of consultations caused by addressing the information in the prevention summary and reminder sheet, or about any process of care issues.

Additionally, the prevention summary and reminder sheets rely on the accuracy and comprehensiveness of the electronic clinical patient data in the practice. While this could be seen as a limitation, it also highlights another benefit of the sheets, namely that they helped to identify inaccurate or missing data, and prompted the patient to raise these instances with the GP. This may contribute toward better quality patient record keeping.

## Conclusions

The positive findings from this study have demonstrated that prevention summary and reminder sheets are perceived by patients as useful aids, which better enable them to discuss their preventive care needs with their GP during consultations. The generalisability of this intervention needs to be explored in a larger study using a cluster randomised trial that will measure changes in actual performance of preventive services and effects on other aspects of care, and a process evaluation that includes feedback from patients, doctors and practice staff. Further studies are also indicated to examine possible refinements to the presentation of information and advice in the prevention summary and reminder sheets to make them as effective as possible.

## Competing interests

The authors declare that they have no competing interests.

## Authors' contributions

ORF conceived the intervention and the study. NPS and PA advised about study design and data collection. PA and ORF analysed the data. ORF drafted the manuscript, with input from NPS and PA. All authors read and approved the final manuscript.

## Pre-publication history

The pre-publication history for this paper can be accessed here:

http://www.biomedcentral.com/1471-2296/12/40/prepub
